# Impacts of accelerating agricultural R&D transfer on global food security

**DOI:** 10.1080/21645698.2024.2438419

**Published:** 2024-12-09

**Authors:** Yan Jin, Zuzana Smeets Kristkova, Maximilian Kardung, Justus Wesseler

**Affiliations:** a Agricultural Economics and Rural Policy Group, Wageningen University, Wageningen, Netherlands; b Department of International Policy, Wageningen Economic Research, The Hague, Netherlands; c Faculty of Economics and Management, Czech University of Life Sciences, Prague, Czech Republic

**Keywords:** Agricultural R&D transfer, computable general equilibrium model, cost of delay, genome editing, global food security

## Abstract

Postponing the adoption of genome editing (GE) is costly, with lengthy regulatory processes contributing to postponement. Accelerating agricultural research and development (R&D) transfer is important for stimulating sustainable agricultural transitions and enhancing global food security. Using the MAGNET model, we incorporate dynamic R&D accumulation and compare economic projections in scenarios with accelerated R&D transfer. We calculate the cost of delay (COD) from postponing GE adoption. The results show that accelerating R&D transfer in high-income countries impacts economic performance, welfare, and food affordability globally; the annuity of COD ranges from losses of -$1.1 billion (Brazil) to gains of $18.5 billion (Europe). A 3-year acceleration of R&D transfer in all countries benefits middle and low-income countries the most (e.g. China, India, other Asian countries, and Sub-Saharan African countries), with the annuity of COD ranging from -$4.8 billion (Brazil) to $83.9 billion (China). Therefore, streamlining the GE regulatory framework is essential for enhancing food security and global welfare.

## 1. Introduction

Research and development (R&D) investment is important for technological innovation and economic growth.^1^ Increasing R&D investment can enhance national security and international competitiveness.^2^ In addition, undertaking R&D and transferring R&D knowledge can promote resilience, prosperity, and economic and social well-being.^3^ Genome editing (GE) is a kind of R&D with broad applications in medicine, microbial engineering, environmental science, and animal and plant breeding.^4^ For example, GE can be used to precisely modify plant genomes and accelerate the development of new crop varieties with increased pest resistance, herbicide tolerance, and biotic and abiotic stress-tolerant agronomical traits (e.g., increased yield) and quality traits (e.g., improved nutritional profile).^5^ However, stringent regulatory processes are hindering the adoption of GE (e.g^6,7^).

The developed products based on the technology must be commercialized and adopted for R&D investments in GE to boost agricultural productivity effectively. Furthermore, it may take years for R&D investments to take effect throughout the gestation, blossom, and obsolete periods.^8^ Studies have shown that knowledge stocks, which accumulate over time due to R&D investment, have a positive impact on technical change^9^ and agricultural productivity growth.^10,11^ However, despite the considerable potential benefits related to knowledge stocks, their impacts are often delayed due to lags caused by the regulatory process for approving new crop varieties.^12^ It has been reported that approval lag due to regulatory processes and high R&D costs hampers productivity growth.^13^ Kalaitzandonakes et al.^14^ estimated that biotechnology-driven crop improvement would take an average of 8–9 years and cost US$22–31 million for a single trait with the current regulatory compliance measures in place for genetically modified organisms (GMOs). Recently, however, the German Research Foundation,^15^ for example, proposed measures to optimize administrative structures and increase the efficiency of GE R&D without lowering protection standards or changing regulations.

A number of studies have been published on the costs of delaying R&D transfer (e.g^16–20^). Most studies focus on a specific crop or a country from an ex-post perspective. Less is known about the implications of accelerating the R&D transfer using a general equilibrium model. To bridge knowledge gaps, we use the Modular Applied GeNeral Equilibrium Tool (MAGNET) to assess the global impacts of accelerating agricultural R&D transfer by comparing projections of macro indicators that result from a scenario in which the status quo is maintained and scenarios in which the regulatory process for GE is simplified. Here, we incorporate a dynamic accumulation of agricultural R&D investment in MAGNET, including region-specific time lags and their links to agricultural productivity. We hypothesize that an acceleration in agricultural R&D transfer positively impacts food security. Literature has shown that promoting agricultural R&D investment can strengthen food security (e.g^21–24^) mainly through technical advancement.^9–11^ Our study aims to test the hypothesis by performing a macroeconomic impact assessment using the MAGNET model.

The rest of the paper is structured as follows. The next section introduces the MAGNET model, its baseline, and scenario design for the impact assessment. In section 3, we present the results: the annuity of COD of postponing adopting GE worldwide and various economic indicators, showing the impacts of accelerating agricultural R&D transfer on global food security. The paper discusses and concludes in section 4.

## 2. Methods

### 2.1. Model Description

MAGNET is a neo-classical, recursive dynamic, multi-sector, multi-region computable general equilibrium (CGE) model that has been used in various policy assessment studies (e.g^25–28^). It is an extension of the Global Trade Analysis Project (GTAP) model, widely adopted for analyzing global trade policies and economic growth by simulating the interactions among countries, industries, and households in a global economic framework. MAGNET is driven by changes in input and output prices, allocating the competing use of primary factors, intermediate inputs, and income and demand responses.^29^ We use MAGNET to perform the impact assessment in this study because it is a global CGE model with a special module for agricultural R&D investment, which enables assessing the economic and food security impacts of a hypothetical agricultural R&D acceleration in different vintage groups of countries (see Table 1 further down) and showing the heterogeneous impacts.

**Table 1.** Parameters for calculating the accumulated knowledge stocks.

	Vintage group	*T*	λ	δ	*g*_*1*_	*g*_*2*_
A	USA, Canada, Australia, and New Zealand	35	0.7	0.8	1.5	3
B	EU-15 and other high-income countries	25	0.6	0.85	5	3
C	EU-12 and former Soviet republics	15	0.4	0.8	0	3
D	Latin America	25	0.7	0.9	0	3
E	Asia, Pacific, and Africa	15	0.5	0.8	0	3

Source: Authors’ elaboration based on Garcia-Alonso et al.^30^, Gbashi et al.^31^, Kristkova et al.^32^, Mmbando^33^, and Yankelevich and Mcgrath^34^.

Note: *g*_*1*_ indicates the approval lag in adopting genome editing (GE) in high-income countries; *g*_*2*_ indicates a hypothetical 3-year approval lag in adopting GE in all countries.

MAGNET data is based on the GTAP database version 10.^35^ The database includes detailed information on production, gross bilateral trade flows, transport costs, and trade protection data for a 2014 benchmark year. The original GTAP database is disaggregated to include additional agricultural and bioeconomy sectors in MAGNET. This study further extends the dataset for the global agricultural R&D investment built from the historical time series of 1960 to 2019 (see appendix p 10 for details). As a result, the MAGNET database contains 67 sectors and 81 commodities.

**Figure 1.**
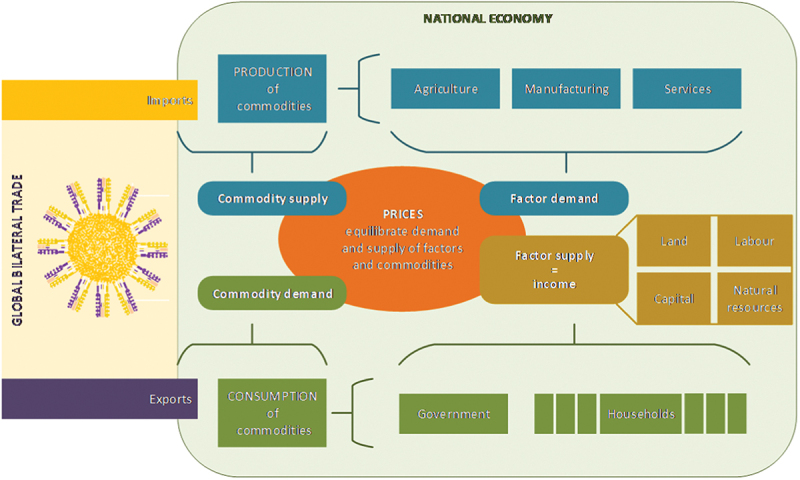
Economic-wide circular flow of goods and services in the MAGNET model – an economic model of nations in the global economy.

Source: MAGNET team.^36^

Figure 1 provides an economic-wide circular flow of goods and services in MAGNET. Every region in the model has a single representative household demanding consumption goods (including savings) on behalf of the private household and the government. Detailed information for the 141 regions can be found in the appendix (S2 pp 8–9). The representative firm creates final domestic products using production factors and intermediate goods under specific technology. A portion of these products enters the domestic market to meet internal demand, and the rest is exported internationally. Total demand is determined by income earned by land, labor, capital, natural resources, and income from taxes. National producers or importers can meet the demand for domestic goods. Households and the government ultimately gain disposable income by employing production factors they own (e.g., labor) and from taxation. They use this income for their consumption and allocate a portion to savings, which is then invested to meet the total domestic demand.

Assuming perfectly competitive markets, producers aim to maximize profits by minimizing costs within specific production technology constraints. Given a certain income level, households maximize their utility based on their preferences. This results in the optimal allocation of supply and demand for factors during the production process. The appendix provides a more detailed model description.

### 2.2. Baseline and Scenario Design

In the last decade, CGE models have established importance in long-term projections based on foresight studies, such as those of the Shared Socioeconomic Pathways (SSPs).^37^ SSPPs are scenarios of possible socioeconomic futures to explore the implications of climate change. They comprise five different narratives of the world’s future with quantified drivers of population, economic activity, urbanization, and income inequality.^38^ The MAGNET model implements the set of SSP scenarios to provide long-term projections of economic development.^28,39–41^

We adopt the SSP2 scenario, which reflects a business-as-usual future based on plausible and alternative assumptions describing future socioeconomic developments in the absence of climate policies or climate change up to 2050, except for those already priced up to the year 2020.^37^ The baseline drivers, assumptions, and data sources used to construct this baseline scenario are described in detail in the appendix (pp 1–5). In the baseline, it is assumed that agricultural R&D investment growth in the future (2020–2050) follows the historical pattern observed over the previous two decades. To make use of the most recent R&D data, the first solution period of MAGNET targets R&D historical growth rates observed in the period 2014–2019.

In this study, we examine improvements in agricultural productivity in two scenarios. In the first scenario, agricultural R&D transfer is accelerated in high-income countries. In the second scenario, it is accelerated in all countries. We implement these counterfactual scenarios and simulate the impacts on selected economic and food security indicators in the mid- and long-term projections for 2030 and 2050, respectively.

In the counterfactual scenarios, all assumptions and drivers mentioned in Table 1 are held the same, except for the gamma distribution (detailed in the next section) of the agricultural R&D transfer. In Scenario 1, we only implement accelerated agricultural R&D transfer in high-income countries. We assume their advanced technological capacity and effect on the global food market as key players. The gamma distribution is modified to mimic accelerated transfer using the approval lag associated with GMOs. It is a hypothetical shock that high-income countries, including the European Union (EU), accelerate GE adoption. This scenario is used to estimate the foregone benefits. However, in reality, we would not expect that the EU would simplify the regulatory process.^a^^a.^We cannot expect that the EU would simplify the regulatory process, especially after the decision of the Judgment of the Court on 25 July 2018^50^ that new plant breeding technologies, including genome editing, will be regulated under the strict regulation of GMOs, subjecting them to extensive safety assessments, traceability, and labeling requirement.^51^ However, a study acknowledged the possible negative impacts of the court decision on public and private research.^52^ Subsequently, the European Commission proposed a new legislative framework,^53^ that classifies plants derived from new genomic techniques into two categories. Category 1 plants, created through targeted mutagenesis or cisgenesis and resembling naturally occurring or conventionally bred plants, are exempt from GMO legislation provided they meet certain criteria. In Scenario 2, the gamma distribution of the agricultural R&D transfer is modified to mimic a 3-year acceleration in all countries. We evalate the impacts of these modifications by comparing economic indicators among the scenarios and baseline conditions.

### 2.3. Methods for Estimating Change in Agricultural Productivity

In both scenarios, the adoption of GE is simplified, which impacts agricultural productivity. The change in agricultural productivity parameter is calculated from the accumulated R&D stock with faster transfer (appendix pp 16–17). We build the accumulated R&D stock from the public agricultural R&D investment linked to the constant elasticity of substitution (CES) production function in MAGNET (appendix pp 6–7). The impacts of the R&D stock are assumed to initially increase in importance, as represented by the increasing weights. Later, the impacts are assigned decreasing weights, approaching zero over time.^42^ Therefore, following the literature,^32^ we assume a maximum lag of *T* years following the gamma distribution to build the knowledge stock for five vintage groups summarized in Table 1.

Following Alston et al.,^43^ we calculate the R&D stock based on Equation 1:

[1] $$\mathrm{RD}\;\mathrm{stoc}k_{i,t}=\sum_{k=0}^{T}b_{k}*R_{i,t-k}$$

where$b_{k}=\frac{{\left(k-g+1\right)}^{\frac{\delta }{1-\delta }}\lambda^{\left(k-g\right)}}{\sum_{k=0}^{T}{\left(k-g+1\right)}^{\frac{\delta }{1-\delta }}\lambda^{\left(k-g\right)}}$, with $\sum_{k=0}^{T}b_{k}=1$

$\mathrm{RDstoc}k_{i,t}$ represents the accumulated knowledge stock per country *i* in year *t*. $R_{i,t-k}$ represents the public R&D investment per country *i* in lag period *t-k*. $b_{k}$ indicates gamma weights that are normalized and sum to one. *T* is the maximum lag of the distribution. λ and δ are gamma distribution parameters that determine the shape of the distribution (0≤λ<1 and 0≤δ<1). The choice of the optimal values for λ and δ in each vintage group depends on the parameters that best fit the data.^9,13^ δ indicates the sensitivity to the timing of the approval, with a higher δ value, implying more sustained reactions over time. λ is a decay parameter that influences how prolonged the approval effects are on the outcome. *g* is the approval lag in adopting GE due to the regulatory process.

In Scenario 1, we approximate the time taken to adopt GE from the reported time to approve GMOs in high-income countries (Vintage Groups A and B).^30,34^ We assume that simplifying the approval process would result in the high-income countries in Vintage Groups A and B adopting GE 1.5 years and 5 years faster, respectively (see Figure A2 in the appendix, p 3). In Scenario 2, we assume a homogenous 3-year faster adoption in all regions, which is based on the world average of regulatory delay.^30,31,33,34,44^ Table 1 summarizes the parameters used to calculate the knowledge stock for each vintage group from the historical (1960–2019) public agricultural R&D investment data.

### 2.4. Methods for Estimating the Cost of Delay

We incorporate the R&D sector as a new production sector and iterate the MAGNET model to 2050 with a recursive dynamic capital stock accumulation for reference calibration. “Recursive” indicates that each time step depends on the results of the previous one.

We define the COD of agricultural R&D transfer as the *N*-year net present value (NPV) of the difference in the gross domestic product (GDP) between each scenario and the baseline, representing the present value of hypothetically simplifying the regulatory process. *N* is the total number of periods (10 years between 2020 and 2030 and 30 years between 2020 and 2050). The NPV equation is shown below.

[2] $$\mathrm{NPV}=\sum_{n=0}^{N}\frac{\mathrm{GD}P_{S,n}-\mathrm{GD}P_{B,n}}{{\left(1+r\right)}^{n}}$$

*n* indicates the individual period. $\mathrm{GD}P_{S,n}-\mathrm{GD}P_{B,n}$ indicates the difference between the GDPs projected in each scenario and at baseline in the *n*^th^ period. *r* is the discount rate, and we set it to 3% in the main text.^18^ Sensitivity analyses are performed using the different discount rates listed in the appendix (pp 11–13).

The annuity of COD – the constant payment each period – is calculated using Equation 3.

[3] $$\mathrm{Annuity}=\frac{\mathrm{NPV}*r}{1-{\left(1+r\right)}^{-N}}$$

This approach underestimates the overall benefits of accelerating the R&D transfer, as it only calculates average annual costs for the next 10 and 30 years.

## 3. Results

The projections of economic indicators calculated for 2050 follow similar patterns to those for 2030; however, the magnitude of the calculated values differs slightly. To avoid repetition, the results of the 2050 projections are presented in the appendix (pp. 13–15).

Tables 2 and 3 show that in the two scenarios, the GDP increases worldwide, except in Brazil and other parts of Latin America. In Scenario 1, the largest increase in GDP happens in EU27, followed by the rest of Europe, Australia, and New Zealand. These are the high-income countries hypothetically accelerating the agricultural R&D transfer. They benefit from adopting GE, which is reflected by the economic indicator of GDP. In Scenario 2, the largest increase in GDP happens in Asia, especially India, as well as Sub-Saharan Africa. Our results indicate that adopting GE will increase productivity (Section S6 in the appendix, pp 16–17) and that more can be produced with the same production factors, stimulating economic growth. The world average GDP increases by 0.04% and 0.19% in the two scenarios may appear modest; however, compared with the baseline of 3.1% in 2030, these gains account for 1.3% and 6.2% of the annual GDP growth in both scenarios. For the EU27, accelerating agricultural R&D transfer contributes 11.7% and 10.4% to GDP growth in the two scenarios.

**Table 2.** Percentage change in indicators by region in scenario 1 (high-income countries shocked), percentage difference compared to the baseline in 2030.

	CAN	USA	BRA	OSA	FSU	EU27	REU	MENA	SSA	CHN	IND	SEA	OAS	ANZ	World
GDP (M$)	0.01	0.01	−0.05	−0.03	0.03	0.18	0.09	0.01	0.02	0.01	0.05	0.00	0.05	0.06	0.04
Welfare (M$)	0.21	0.14	−0.05	−0.10	−0.05	1.81	1.00	−0.09	−0.03	−0.04	−0.02	−0.01	−0.01	0.75	0.30
Land rent (M$)	−14.6	−7.0	−3.8	−6.7	−9.9	−23.8	−19.4	−9.9	−4.0	−3.7	−2.4	−2.3	−2.7	−9.7	−5.2
Per capita per day quantity of calories (kcal)	0.5	0.7	0.0	0.2	0.4	1.2	1.0	1.1	0.4	0.2	0.3	0.1	0.8	0.8	0.6
Unskilled wages to the cereal price index	5.13	3.11	−0.61	0.88	5.36	11.75	11.89	3.13	1.08	1.02	1.81	0.06	2.54	5.64	2.30

Note 1: M$ indicates million US dollars; kcal indicates kilocalorie.

Note 2: CAN=Canada, USA=United States, BRA=Brazil, OSA=Other Latin America, REU=Other Europe apart from EU27, MENA=Middle-East and North Africa, SSA=Sub-Saharan Africa, CHN=China, IND=India, SEA=South-East Asia, OAS=Other Asia, ANZ=Australia and New Zealand.

**Table 3.** Percentage change in indicators by region in scenario 2 (all countries shocked), percentage difference compared to the baseline in 2030.

	CAN	USA	BRA	OSA	FSU	EU27	REU	MENA	SSA	CHN	IND	SEA	OAS	ANZ	World
GDP (M$)	0.03	0.02	−0.10	−0.04	0.21	0.16	0.07	0.18	0.68	0.33	0.74	0.11	0.87	0.14	0.19
Welfare (M$)	0.42	0.29	0.02	−0.12	1.00	1.33	0.71	1.26	2.84	1.43	2.29	1.23	3.13	1.43	1.15
Land rent (M$)	−30.8	−16.2	−8.1	−12.8	−18.0	−25.4	−23.1	−19.4	−17.0	−27.7	−15.5	−8.7	−15.0	−26.0	−18.0
Per capita per day quantity of calories (kcal)	1.9	2.2	0.3	0.6	1.9	1.8	2.0	2.0	2.2	2.7	2.3	0.9	1.7	2.4	1.8
Unskilled wages to the cereal price index	9.35	6.73	−1.13	2.04	11.19	8.49	11.01	8.41	10.85	16.18	15.20	6.04	16.34	11.57	10.39

Note 1: M$ indicates million US dollars; kcal indicates kilocalorie.

Note 2: CAN=Canada, USA=United States, BRA=Brazil, OSA=Other Latin America, REU=Other Europe apart from EU27, MENA=Middle-East and North Africa, SSA=Sub-Saharan Africa, CHN=China, IND=India, SEA=South-East Asia, OAS=Other Asia, ANZ=Australia and New Zealand.

Compared with GDP reflecting a country’s total economic output, welfare is measured using the equivalent variation. Equivalent variation is a metric that assesses the change in consumer satisfaction or utility. Our results suggest that welfare can be affected by changes in production and price, as welfare is impacted by trade (Tables 4 and 5). In Scenario 1, technological improvements lead to increased welfare in high-income countries but slight decreases in welfare in middle and low-income countries. In Scenario 2, welfare increases in all countries except for those in Latin America. Due to the increased agricultural productivity, the same acreage of land can be used to produce more efficiently; therefore, land becomes a relatively less scarce resource, and the rental price of land decreases worldwide in both scenarios. In Scenario 1, the largest decrease in land rent happens in EU27 and other parts of Europe. This is due to the large agricultural productivity increase in those regions (Section S6 in the appendix, pp 16–17). In Scenario 2, the largest decrease in land rent happens in Canada, Europe, China, Australia, and New Zealand, with a percentage change reduction of more than 20%.

**Table 4.** Percentage change in trade volume indicators by region in scenario 1 (high-income countries shocked), percentage difference compared to the baseline in 2030.

		CAN	USA	BRA	OSA	FSU	EU27	REU	MENA	SSA	CHN	IND	SEA	OAS	ANZ
Export volume	Primary agriculture	0.2	0.9	−7.1	−7.0	−5.2	35.8	32.9	−14.7	−9.3	−9.9	−10.7	−9.1	−8.5	3.1
	Food processing	−0.1	−1.2	0.2	−1.2	−0.7	4.9	1.5	0.8	−2.3	−2.2	−1.1	−1.3	−1.5	2.3
	Aggregated agri–food	0.0	−0.3	−3.9	−4.4	−3.0	11.0	4.5	−5.9	−7.4	−4.8	−3.6	−2.7	−2.7	2.6
Import volume	Primary agriculture	1.9	−0.7	2.5	1.6	3.0	−20.8	−5.2	4.7	8.8	2.1	2.5	1.6	2.9	−2.4
	Food processing	0.3	1.0	−0.3	1.2	1.9	−3.1	0.5	0.6	1.8	2.2	0.8	1.5	0.7	0.9
	Aggregated agri–food	0.8	0.4	0.6	1.3	2.3	−11.0	−1.0	2.4	2.7	2.1	1.5	1.6	1.7	0.4

Note 1: CAN=Canada, USA=United States, BRA=Brazil, OSA=Other Latin America, REU=Other Europe apart from EU27, MENA=Middle-East and North Africa, SSA=Sub-Saharan Africa, CHN=China, IND=India, SEA=South-East Asia, OAS=Other Asia, ANZ=Australia and New Zealand.

Note 2: Trade flows in Table 4 refer to aggregated trade to the rest of the world, excluding intra-regional trade.

**Table 5.** Percentage change in trade volume indicators by region in scenario 2 (all countries shocked), percentage difference compared to the baseline in 2030.

		CAN	USA	BRA	OSA	FSU	EU27	REU	MENA	SSA	CHN	IND	SEA	OAS	ANZ
Export volume	Primary agriculture	−0.4	−0.5	−14.6	−11.7	5.6	7.2	9.9	−7.2	6.7	21.2	11.0	−6.6	15.4	4.0
	Food processing	−0.9	−4.0	−1.5	−4.2	−1.0	1.0	−1.9	4.4	−0.1	10.5	15.4	1.1	0.4	3.5
	Aggregated agri–food	−0.7	−2.5	−8.9	−8.3	2.4	2.2	−0.8	−0.5	4.9	14.0	14.2	−0.3	3.0	3.7
Import volume	Primary agriculture	3.8	−1.9	5.0	4.0	−1.2	−7.5	−3.2	0.8	0.7	−6.0	−3.0	2.7	−3.7	−5.9
	Food processing	0.7	2.2	−0.8	2.7	1.6	1.1	2.0	−0.7	2.7	−3.9	−1.7	1.7	−2.6	1.6
	Aggregated agri–food	1.6	0.7	1.2	3.2	0.4	−2.8	0.6	−0.1	2.4	−5.2	−2.2	2.1	−3.1	0.3

Note 1: CAN=Canada, USA=United States, BRA=Brazil, OSA=Other Latin America, REU=Other Europe apart from EU27, MENA=Middle-East and North Africa, SSA=Sub-Saharan Africa, CHN=China, IND=India, SEA=South-East Asia, OAS=Other Asia, ANZ=Australia and New Zealand.

Note 2: Trade flows in Table 5 refer to aggregated trade to the rest of the world, excluding intra-regional trade.

Surplus gains due to increased productivity lead to higher caloric supply worldwide, especially in Scenario 2 (global acceleration of GE adoption). In Scenario 1, the largest increase in per capita per day quantity of calories happens in EU27, other parts of Europe, and Middle-East and North Africa. For Middle-East and North Africa, this is mainly due to a large reduction in exports and an increase in imports (See Table 4). In Scenario 2, the largest increase in per capita per day quantity of calories happens in China, India, and the USA, mainly due to increased production. In both scenarios, the ratio of unskilled wages to the cereal price index increases worldwide, indicating a jump in food affordability,^32^ except in Brazil, due to lower cereal production and higher prices. In Scenario 1, the largest increase in unskilled wages to the cereal price index happens in EU27 and other parts of Europe, with a change of more than 11%. In Scenario 2, the largest increase in unskilled wages to the cereal price index happens in middle and low-income countries like China, India, and other Asian countries, mainly due to lower cereal prices resulting from increased production.

Tables 4 and 5 illustrate the exports and imports of the primary agricultural production, food processing, and agri-food aggregated (production plus processing) stages. As shown in Table 4, the major change in export and import volume occurs in primary agricultural production compared with food processing. During the aggregated stage, the EU27 has the most prominent export increase compared with the rest of the world in Scenario 1, the largest beneficiary among the high-income countries; India and China have the most significant increase in exports compared with the rest of the world in Scenario 2. Their potential export opportunities mainly result from the increased agricultural production.

The EU27 shows the largest decline in import volume at the aggregated stage. This suggests that the EU increases its self-sufficiency regarding the domestic supply of primary agricultural commodities, as increased productivity leads to greater domestic production. In Scenario 1, imports increase in other parts of the world to absorb the EU’s agricultural surplus. The EU27 is the largest beneficiary among the high-income countries. In Scenario 2, increased production reduces imports in large regions like China, other Asian countries, India, and EU27.

Figures 2a and 3a show the COD between 2020 and 2030 due to slow agricultural R&D transfer. In Scenario 1, the EU27 ($158 billion), the rest of Europe ($23 billion), and the USA ($13 billion) have the largest COD. In contrast, in Scenario 2, China ($358 billion), India ($147 billion), and the EU27 ($135 billion) have the largest COD. However, in both scenarios, Brazil and other Latin American countries have a negative COD, indicating that they benefit from delaying the adoption of GE due to their current cultivation and export of genetically modified crops. In Scenario 1, most of the world loses economically due to the delay, with the total combined COD of all countries up to $215 billion between 2020 and 2030; in Scenario 2, the total combined COD of all countries is up to $983 billion.

**Figure 2a.**
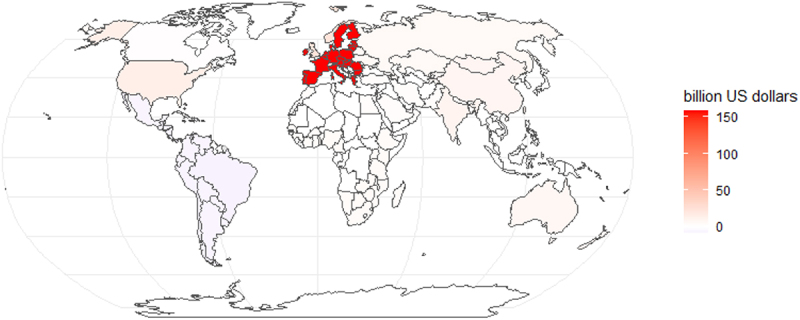
Cost of delay (COD) between 2020 and 2030 (high-income countries shocked).

Note: The COD was calculated using a discount rate of 3%. Sensitivity analyses of the COD based on different discount rates are included in the appendix (pp 11–13).

**Figure 3a.**
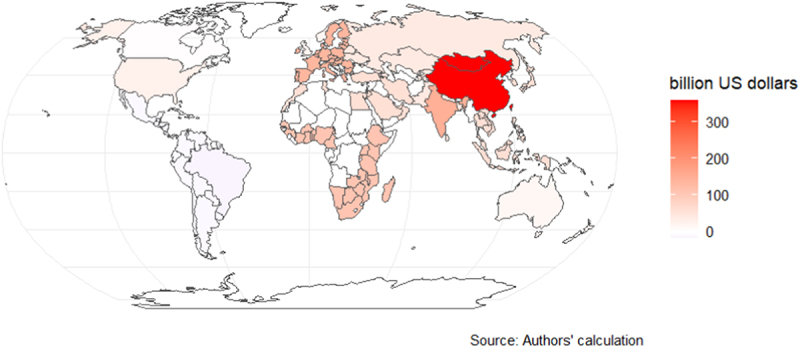
Cost of delay (COD) between 2020 and 2030 (all countries shocked).

Note: The COD was calculated using a discount rate of 3%. Sensitivity analyses of the COD based on different discount rates are included in the appendix (pp 11–13).

The annuity follows a similar pattern to the COD, as shown in Figures 2b and 3b. In Scenario 1, the annuity ranges from losses of -$1.1 billion (Brazil) to gains of $18.5 billion (EU27), and in Scenario 2, the annuity ranges from -$4.8 billion (Brazil) to $83.9 billion (China).

**Figure 2b.**
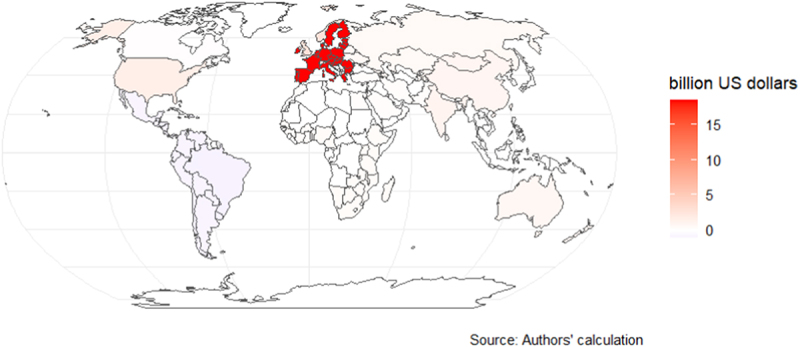
Annuity between 2020 and 2030 (high-income countries shocked).

**Figure 3b.**
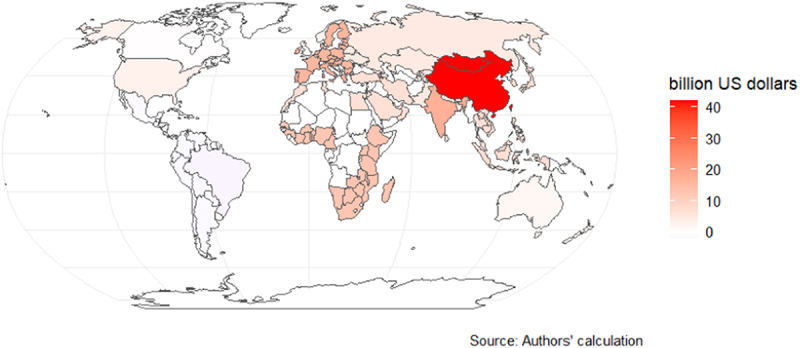
Annuity between 2020 and 2030 (all countries shocked).

## 4. Discussion and Conclusion

In this study, we examine the impacts of accelerating agricultural R&D transfer and estimate the COD of adopting GE between 2020 and 2030 (the COD between 2020 and 2050 is detailed in the appendix, pp 11–13). Our results show that accelerating agricultural R&D transfer in high-income countries directly impacts their economic performance, welfare, input cost, nutrients, food affordability, and a spillover effect on middle and low-income countries. Therefore, simplifying the GE approval process benefits most countries except Brazil. We also find that China, India, other Asian countries, and Sub-Saharan African countries benefit the most from a homogenous 3-year increase in agricultural R&D transfer across all countries. Furthermore, our results indicate that accelerating agricultural R&D transfer increases agricultural productivity and, consequently, producer surplus, consumer surplus, and caloric consumption. It is worth noting that in both scenarios, we examine Brazil – the world’s largest trader of genetically modified crops – economically loses the most. It should also be highlighted that the EU27 is likely to be the biggest beneficiary of accelerated agricultural R&D transfer in high-income countries. Middle and low-income countries, such as China and India, will likely be the largest beneficiaries of a global acceleration in agricultural R&D transfer.

Several limitations of this study should be noted. First, we assume no ban on GE commercialization. However, the situation is complex and involves various stakeholders and lobby groups, some of which favor a ban. Second, this study is conducted at a macro level with aggregated data and does not consider the heterogeneity of individual farms or farm types. To overcome this limitation, future studies could link macro modeling with micro simulations to explore the heterogeneity of the impacts on different types of farms. Third, the agricultural R&D dataset we use for the analysis covers the period between 1960 and 2019, the latest global compatible R&D dataset available. However, it does not cover the most recent years where the R&D investment patterns in individual countries may have changed over time. Fourth, our study only assesses the direct impacts of accelerating agricultural R&D transfer without considering the potential indirect impacts, such as the spillover effects of adopting GE. Another potential indirect channel is the increased caloric intake and improved health effects of adopting GE using the methodology of disability-adjusted life years (e.g.^45–47,48^). Therefore, the results of this study need to be taken with caution as it is likely to be on the conservative side since the indirect impacts of accelerating agricultural R&D transfer are not explicitly taken into account, but they can be substantial. In addition, as Brookes and Barfoot^17^ and Klümper et al.^49^ have shown, environmental benefits can also be substantial. Last, it is worth mentioning that advances in plant genetics are one type of agricultural R&D that can affect agricultural production systems. In addition, although both public and private R&D make significant contributions to agricultural productivity,^11^ we only assess the impacts of accelerating public R&D transfer on global food security. Therefore, it can be considered a conservative estimation of the impacts without considering the contribution from the private sector. There are two-way dynamic effects between public and private R&D,^11^ and future research is encouraged to dig into the private sector and its interactions between the public and private sectors once data become available.

In summary, despite the above uncertainties, our findings provide valuable insights into the impacts of accelerating agricultural R&D transfer and the estimated COD of adopting GE. The absolute magnitude of the effects could vary; however, the same patterns are observed in the sensitivity analyses. As we recognize the substantial benefits currently being forfeited, we recommend that policymakers streamline the regulatory process for adopting GE, which has been proven safe and is ready for commercialization, to enhance food security and global welfare.

## Supplementary Material

Appendix_revision.docx

## Data Sharing

Data sources are listed in Table A3 in the appendix (p 10). Additional data can be shared upon reasonable request to the authors.
