# Supplementary material for: Impacts of accelerating agricultural R&D transfer on global food security
Source: GM Crops Food. 2024 Dec 9;15(1):1–12. doi: 10.1080/21645698.2024.2438419 (PMC11633200; doi:10.1080/21645698.2024.2438419)
Supplement: Appendix_revision.docx [file KGMC_A_2438419_SM5168.docx]

Supplementary information for

**Impacts of Accelerating Agricultural R&D Transfer on Global Food Security**

**S1. The MAGNET model**

S1.1 Detailed model description

Modular Applied GeNeral Equilibrium Tool (MAGNET) is an extension of the Global Trade Analysis Project (GTAP) model, widely adopted for global trade analysis. MAGNET is a neo-classical recursive dynamic multi-sector, multi-region computable general equilibrium (CGE) model driven by changes in input and output prices allocating the competing use of primary factors, intermediate inputs, and income and demand responses (Woltjer et al., 2014). MAGNET is suitable for impact assessment in the medium-to-long run. MAGNET assumes that producers are perfect competitors and exhibit constant returns to scale technology and zero long-term economic profits. By solving the supply, demand, and price system of various interacting factors and sectors reflecting the global economy, MAGNET finds general equilibrium solutions under the clearing conditions that supply equals demand, the value of income from production factors, expenditures, and output are equal, and the net balance between the current account, i.e., export-import difference, and the capital account, i.e., saving-investment difference, amounts to zero (De Jong et al., 2023).

The flow of factors, goods, and services in the economic system in the MAGNET has been documented in detail (MAGNET team, 2024). Figure A1 provides a summary. Every region in the model has a single representative household demanding consumption goods (including savings) on behalf of the private household and the government. The representative household creates final domestic products under specific technical conditions using production factors and intermediate goods. A portion of these products enters the domestic market to meet internal demand, and the rest is exported internationally. Total demand is determined by income earned by land, labor, capital, natural resources, and income from taxes. National producers or imports can meet the demand for domestic goods. Households and the government ultimately gain disposable income by selling factors and taxation. They use this income for their consumption and allocate a portion to savings, which is then invested to meet the total domestic demand. Assuming perfectly competitive markets, producers aim to maximize profits by minimizing costs within specific production technology constraints. Given a certain income level, households maximize their utility based on their preferences. This results in the optimal allocation of supply and demand for factors during the production process.


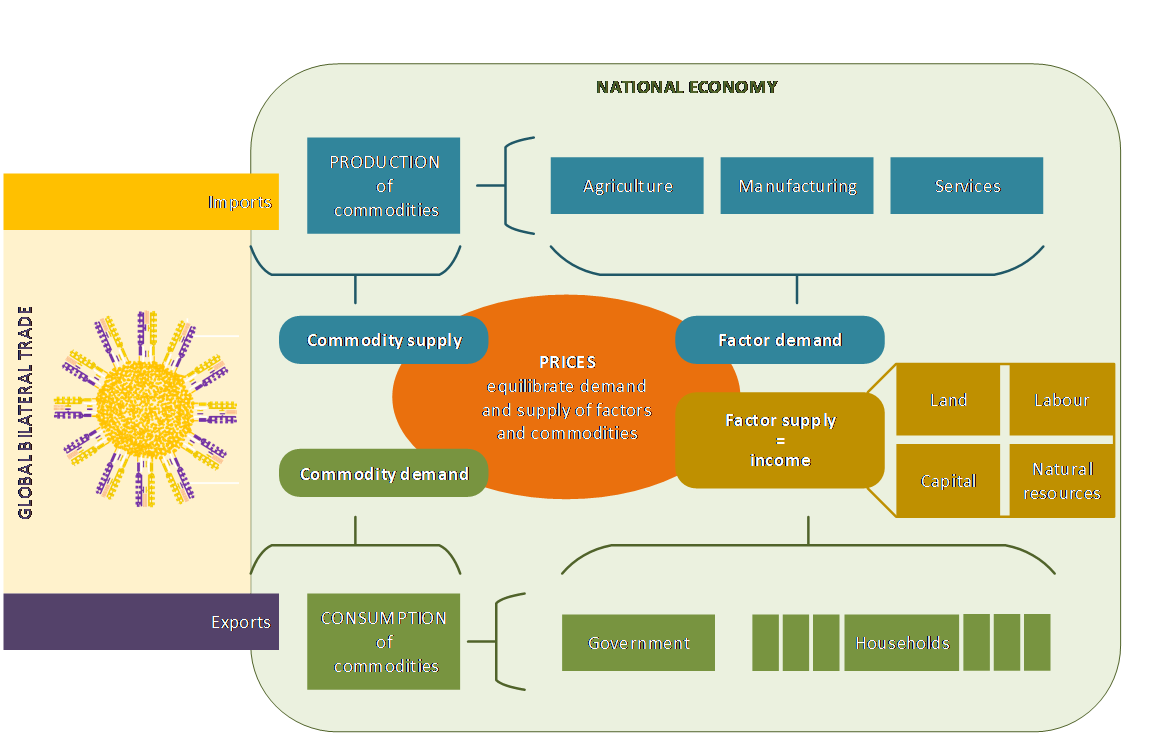


Figure A1. Economic-wide circular flow of goods and services in the MAGNET – an economic model of nations in the global economy

Source: MAGNET team (2024)

MAGNET is built based on the GTAP model and its core data structure with an input-output accounting framework. Compared to the classic GTAP model, one advantage of MAGNET is that the land use is endogenous by including a land supply curve estimated with historical information on land price, supply, and bio-physical information (Van Meijl et al., 2006). Another advantage is its modular structure, which combines various research strands and enables trade-off analysis across various domains. MAGNET has been widely adopted to simulate the impacts of policies related to agri-food, trade, land use, biofuel, and climate in the short, medium, and long run (e.g., Kuiper & Cui, 2021; Kuiper et al., 2020; Frank et al., 2019; Robinson et al., 2014; Philippidis et al., 2020; Van Meijl et al., 2020). Version 10 of the GTAP database with the benchmark year 2014 is used in the current version of MAGNET. It has 113 sectors, 127 commodities, eight primary factors, and 141 regions and countries. All transactions are measured at basic producer and purchaser prices.

In the study, we specifically apply a MAGNET module assessing the impacts of public R&D investments. We focus on the pubic R&D transfer in the agricultural sector, targeting accelerating the adoption of genome editing (GE). Therefore, following the literature (Piesse et al., 2010; Kristkova et al., 2016; Thirtle et al., 2002), we assume that public agricultural R&D investment related to land-oriented research for GE is responsible for agricultural productivity resulting from improved crop varieties. The advantage of linking public agricultural R&D investment to agricultural productivity is that it reflects the characteristics of agricultural R&D investment as a public good that the government pays for the R&D expenditures while the agricultural sector benefits. As introduced in detail in the main text, a gamma distribution with a gestation lag *g* reflects the pattern of agricultural R&D transfer - accumulated R&D stock. The gestation lags of 0 (baseline), 1.5, and 5 years are illustrated in Figure A2.

Figure A2. A gamma distribution with a shift of gestation lag *g* in Equation 1 in the main text.

Note: In Figure A2, *T* = 10, $\lambda$ = 0.7, $\delta$ = 0.85 are used for demonstration.

S1.2 Model assumptions and baseline drivers

Table A1 summarises the baseline drivers and assumptions used in the MAGNET model. Figures A3 and A4 show the Shared Socioeconomic Pathways #2 (SSP2) projections for population and gross domestic product (GDP), respectively.

Table A1. Baseline drivers and assumptions

|  | Description |
| --- | --- |
| Macroeconomic drivers | Population growth: SSP2 – Medium variant |
|  | GDP growth: SSP2 scenario |
| Sectoral productivity drivers | Land productivity: Endogenously based on R&D investments |
|  | Feed efficiency improvements in livestock sectors: 1% – 2% p.a. based on the IMAGE model – An ecological-environmental model framework developed by the Netherlands Environmental Assessment Agency |
| Policy assumptions | CO2 prices in the European Union (a gradual increase from $30/ton to over $110/ton in 2050) |
|  | Public agricultural R&D investment growth follows the historical growth rates in the past two decades. |

Note: An introduction of the IMAGE model can be found here: <https://www.pbl.nl/en/image/home>

Figure A3. SSP2 population projections (% growth rate)

Note: CAN=Canada, USA=United States, BRA=Brazil, OSA=Other Latin America, REU=Other Europe apart from EU27, MENA=Middle-East and North Africa, SSA=Sub-Saharan Africa, CHN=China, IND=India, SEA=South-East Asia, OAS=Other Asia, ANZ=Australia and New Zealand.

Figure A4. SSP2 GDP projections (% growth rate)

Note: CAN=Canada, USA=United States, BRA=Brazil, OSA=Other Latin America, REU=Other Europe apart from EU27, MENA=Middle-East and North Africa, SSA=Sub-Saharan Africa, CHN=China, IND=India, SEA=South-East Asia, OAS=Other Asia, ANZ=Australia and New Zealand.

S1.3 Incorporating the accumulated R&D stock into the MAGNET model

This section introduces how the accumulated R&D stock is incorporated into the MAGNET model via the Constant Elasticity of Substitution (CES) production function.

First, the accumulated R&D stock growth from the gamma distribution is linked to the factor-augmenting technical change in MAGNET shown in Equation A1.

${afa\_RD}_{j,i}=\delta_{RD,i}*{RDstock\_growth}_{i}$ [A1]

where ${afa\_RD}_{j,i}$ indicates the period growth of the aggregated factor-augmenting technical change parameter for sector *j* in country *i*. The matrix of gamma weights for each vintage group derived from ${RDstock}_{i,t}$ are aggregated into four simulation periods in MAGNET (P1: 2014-2020; P2: 2020-2030; P3: 2030-2040; P4: 2040-2050). ${RDstock\_growth}_{i}$ is the annual growth rate of domestic R&D stock per MAGNET region (see Section S2 in the appendix). $\delta_{RD,i}$ is the elasticity of ${afa\_RD}_{j,i}$ with respect to the R&D stock growth.Next, ${afa\_RD}_{j,i}$is fed into a composite factor-augmenting technical change in Equation A2.

${afa}_{e,j,i}=$ ${{ASCALE}_{e,j,i}\cdot{aknreg}_{i}+{DUM_{TC}}_{e,j,i}\cdot afa\_RD}_{j,i}+{DUM\_I\_LAND}_{j}\cdot{aland}_{j,r}$ [A2]

where ${aknreg}_{i}$ refers to an economy-wide factor augmenting technical change obtained as a residual when targeting expected GDP growth. By multiplying with the ${ASCALE}_{e,j,i}$ coefficient, defined for input *e*, sector *j,* and country *i*, the macroeconomic productivity growth is distributed over the production sectors. This results in productivity growth rates that are sector and input-specific. This is the main source of labor productivity growth in agriculture in MAGNET.

The second component is the R&D-driven productivity growth ${afa\_RD}_{j,i}$ obtained from Equation A1. In the default setting, all endowments in agriculture (land, labor, and capital) benefit equally from R&D-driven productivity growth. This is guided by parameter ${DUM_{TC}}_{e,j,i}$ for input *e*, sector *j,* and country *i*.

The remaining component ${aland}_{i,r,}$ contains other possible productivity sources, such as changes in the shares of irrigated water or climate change-specific yields. The coefficient ${DUM\_I\_LAND}_{j}$ ensures only land-using sectors are considered for this source of productivity growth.

Equation A3 lists a simplified version of the CES production function, with the composite factor-augmenting technical change incorporating the accumulated R&D stock via Equations A1 and A2.

${ValueAdded}_{j,i}=\left[ a*\left( {afa}_{L,j,i}*L_{j,i} \right)^{\frac{\sigma_{K,L}-1}{\sigma_{K,L}}}+\left( 1-a \right)*\left( {afa}_{K,j,i}*K_{j,i} \right)^{\frac{\sigma_{K,L}-1}{\sigma_{K,L}}} \right]^{(\frac{\sigma_{K,L}}{\sigma_{K,L}-1})}$ [A3]

where $L_{j,i}$ indicates the land input for sector *j* in country *i* and $K_{j,i}$ indicates the input of the capital-labor bundle. $\sigma_{K,L}$ is the elasticity of substitution between land and capital-labor inputs. $a$ represents the share of each kind of input in the value-added. The composite factor-augmenting technical change ${afa}_{e,j,i}$ defined for endowment *e*, sector *j,* and country *i* includes several sources of factor productivity growth in agriculture.

**S2. Classification of regions in the MAGNET model**

As shown in Figure A5, apart from some large countries, such as Brazil, Canada, China, India, and the United States, the MAGNET aggregates the rest of the world into nine geographic regions, with main developed and developing economies as separate regions. The detailed regional aggregation is summarized in Table A2.


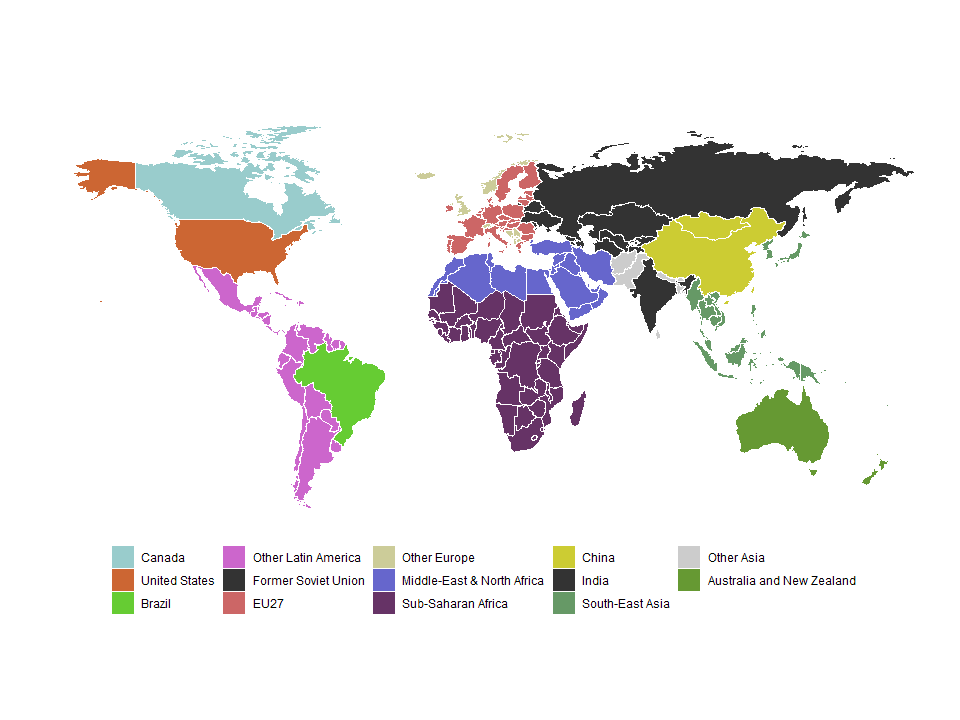


Figure A5. A map of regions represented in the MAGNET

Source: MAGNET team (2024)

Table A2. Regions in the MAGNET

| Region code | Region name | Major countries included |
| --- | --- | --- |
| CAN | Canada | Canada |
| USA | United States | United States |
| BRA | Brazil | Brazil |
| OSA | Other Latin America | Argentina, Chile, Dominican Republic, Mexico, Uruguay, Bolivia, Peru, Colombia, Panama, Costa Rica, Nicaragua, Honduras, El Salvador, Guatemala, Venezuela, Ecuador, Jamaica, Paraguay, Trinidad and Tobago, Puerto Rico |
| FSU | Former Soviet Union | Kazakhstan, Russia, Tajikistan, Kyrgyzstan, Armenia, Belarus, Ukraine, Azerbaijan, Georgia |
| EU27 | EU27 | EU27 |
| REU | Other Europe | United Kingdom, Iceland, Norway, Switzerland |
| MENA | Middle-East & North Africa | Israel, Tunisia, Jordan, United Arab Emirates, Qatar, Kuwait, Oman, Iran, Turkey, Saudi Arabia, Morocco, Egypt |
| SSA | Sub-Saharan Africa | Tanzania, Kenya, South Africa, Zimbabwe, Botswana, Namibia, Senegal, Benin, Nigeria, Cameroon, Togo, Ghana, Ivory Coast, Guinea, Burkina Faso, Zambia, Malawi, Mozambique, Madagascar, Ethiopia, Uganda, Rwanda, Mauritius |
| CHN | China | China, Hong Kong, Taiwan, Mongolia |
| IND | India | India |
| SEA | South-East Asia | Indonesia, Cambodia, Thailand, Laos, Vietnam, South Korea, Philippines, Malaysia, Brunei, Japan |
| OAS | Other Asia | Bangladesh, Nepal, Pakistan, Sri Lanka |
| ANZ | Australia & New Zealand | Australia, New Zealand |

Source: MAGNET team (2024)

**S3. Data sources**

Table A3 summarizes the major data sources of the MAGNET model.

Table A3. Summary of major data sources

| Data | Source |
| --- | --- |
| Input-output and bilateral trade data in 2014 | Global Trade Analysis Project database (GTAP10) |
| Prices for agricultural products | FAOSTAT publication and the International Institute for Applied Systems Analysis (IIASA) database |
| Fertilizer use | Informationsstelle für Arzneispezialitäten (IFA) database |
| Land asymptote | IMAGE model (Netherlands Environmental Assessment Agency) |
| Energy and heat quantities | International Energy Agency (IEA) and Energy Information Administration (EIA) database |

The R&D investment is based on data from EUROSTAT, the Organization for Economic Co-operation and Development (OECD), the United Nations Educational, Scientific and Cultural Organization (UNESCO), Agricultural Science and Technology Indicators (ASTI), Network for Science and Technology Indicators (RICYT), International Service for National Agricultural Research (ISNAR) Agricultural Research Indicators, and national statistics for the period between 1981 and 2019. The MAGNET team harmonized data definitions from various sources and converted them to constant 2014 US dollars to ensure data compatibility. The datasets cover high-income countries. However, for countries and sectors with missing data, imputation techniques were adopted to estimate and approximate (Kristkova et al., 2022).

**S4. Sensitivity analyses of the cost of delay**

We performed sensitivity analyses of the cost of delay (COD) with various discount rates (1%–10%) to demonstrate the robustness of our results. Tables A4–A7 show the COD between 2020–2030 and 2020–2050, respectively.

Table A4. Cost of delay worldwide between 2020 and 2030 with different discount rates (high-income countries shocked)

| Region | COD (1%) | COD (3%) | COD (5%) | COD (10%) |
| --- | --- | --- | --- | --- |
| CAN | 1.6 | 1.4 | 1.3 | 0.9 |
| USA | 15.1 | 13.1 | 11.5 | 8.4 |
| BRA | -10.3 | -8.9 | -7.8 | -5.7 |
| OSA | -8.1 | -7.1 | -6.2 | -4.5 |
| FSU | 6.1 | 5.3 | 4.7 | 3.4 |
| EU27 | 180.8 | 157.7 | 138.2 | 101.5 |
| REU | 26.1 | 22.7 | 19.9 | 14.6 |
| MENA | 2.9 | 2.5 | 2.2 | 1.6 |
| SSA | 4.0 | 3.5 | 3.0 | 2.2 |
| CHN | 9.2 | 8.0 | 7.0 | 5.1 |
| IND | 10.4 | 9.0 | 7.9 | 5.7 |
| SEA | -1.6 | -1.4 | -1.2 | -0.9 |
| OAS | 2.5 | 2.2 | 1.9 | 1.4 |
| ANZ | 7.9 | 6.9 | 6.1 | 4.4 |

Note: CAN=Canada, USA=United States, BRA=Brazil, OSA=Other Latin America, REU=Other Europe apart from EU27, MENA=Middle-East and North Africa, SSA=Sub-Saharan Africa, CHN=China, IND=India, SEA=South-East Asia, OAS=Other Asia, ANZ=Australia and New Zealand.

Table A5. Cost of delay worldwide between 2020 and 2050 with different discount rates (high-income countries shocked)

| Region | COD (1%) | COD (3%) | COD (5%) | COD (10%) |
| --- | --- | --- | --- | --- |
| CAN | 8.1 | 5.5 | 3.9 | 1.9 |
| USA | 82.5 | 58.0 | 42.0 | 21.0 |
| BRA | -113.6 | -77.6 | -54.3 | -24.5 |
| OSA | -53.1 | -37.0 | -26.5 | -12.8 |
| FSU | 58.5 | 38.4 | 26.0 | 11.2 |
| EU27 | 696.7 | 510.0 | 383.6 | 208.7 |
| REU | 100.0 | 73.5 | 55.4 | 30.2 |
| MENA | 59.1 | 38.4 | 25.6 | 10.3 |
| SSA | 37.3 | 26.0 | 18.5 | 8.6 |
| CHN | 14.0 | 17.9 | 17.9 | 13.1 |
| IND | 86.3 | 59.4 | 42.0 | 19.6 |
| SEA | 1.9 | 0.8 | 0.1 | -0.5 |
| OAS | 40.4 | 26.6 | 17.9 | 7.4 |
| ANZ | 38.8 | 27.8 | 20.5 | 10.6 |

Note: CAN=Canada, USA=United States, BRA=Brazil, OSA=Other Latin America, REU=Other Europe apart from EU27, MENA=Middle-East and North Africa, SSA=Sub-Saharan Africa, CHN=China, IND=India, SEA=South-East Asia, OAS=Other Asia, ANZ=Australia and New Zealand.

Table A6. Cost of delay worldwide between 2020 and 2030 with different discount rates (all countries shocked)

| Region | COD (1%) | COD (3%) | COD (5%) | COD (10%) |
| --- | --- | --- | --- | --- |
| CAN | 3.4 | 3.0 | 2.6 | 1.9 |
| USA | 30.4 | 26.5 | 23.2 | 17.0 |
| BRA | -19.2 | -16.7 | -14.6 | -10.7 |
| OSA | -11.2 | -9.8 | -8.5 | -6.2 |
| FSU | 45.8 | 39.8 | 34.8 | 25.4 |
| EU27 | 154.8 | 135.0 | 118.4 | 86.9 |
| REU | 21.5 | 18.8 | 16.4 | 12.1 |
| MENA | 66.0 | 57.4 | 50.2 | 36.6 |
| SSA | 124.3 | 107.9 | 94.2 | 68.4 |
| CHN | 412.5 | 358.4 | 312.9 | 227.5 |
| IND | 170.0 | 147.5 | 128.7 | 93.4 |
| SEA | 68.5 | 59.7 | 52.2 | 38.2 |
| OAS | 47.3 | 41.1 | 35.9 | 26.0 |
| ANZ | 16.8 | 14.6 | 12.8 | 9.4 |

Note: CAN=Canada, USA=United States, BRA=Brazil, OSA=Other Latin America, REU=Other Europe apart from EU27, MENA=Middle-East and North Africa, SSA=Sub-Saharan Africa, CHN=China, IND=India, SEA=South-East Asia, OAS=Other Asia, ANZ=Australia and New Zealand.

Table A7. Cost of delay worldwide between 2020 and 2050 with different discount rates (all countries shocked)

| Region | COD (1%) | COD (3%) | COD (5%) | COD (10%) |
| --- | --- | --- | --- | --- |
| CAN | 4.2 | 3.0 | 2.3 | 1.6 |
| USA | 89.5 | 64.6 | 48.5 | 27.3 |
| BRA | -131.5 | -93.2 | -67.6 | -33.3 |
| OSA | 21.6 | 10.3 | 3.8 | -2.6 |
| FSU | 221.2 | 156.9 | 114.6 | 58.7 |
| EU27 | 349.3 | 276.4 | 222.7 | 138.6 |
| REU | 55.9 | 43.4 | 34.3 | 20.6 |
| MENA | 341.4 | 241.6 | 175.9 | 89.1 |
| SSA | 670.2 | 471.7 | 341.7 | 171.1 |
| CHN | 2313.2 | 1644.9 | 1200.0 | 604.4 |
| IND | 1098.5 | 763.1 | 545.1 | 263.4 |
| SEA | 280.5 | 203.9 | 152.4 | 81.7 |
| OAS | 360.1 | 246.7 | 173.7 | 81.1 |
| ANZ | 72.5 | 52.6 | 39.2 | 20.8 |

Note: CAN=Canada, USA=United States, BRA=Brazil, OSA=Other Latin America, REU=Other Europe apart from EU27, MENA=Middle-East and North Africa, SSA=Sub-Saharan Africa, CHN=China, IND=India, SEA=South-East Asia, OAS=Other Asia, ANZ=Australia and New Zealand.

**S5. Results based on the projections for 2050**

This section presents the long-term impact based on the projections for 2050. The main text describes the short-term impact based on the projected indicators in 2030. The projected indicators in 2050 follow similar patterns to those in 2030 but with some differences in the magnitude of the calculated values.

Table A8a. Percentage change in indicators by region in Scenario 1 (high-income countries shocked), percentage difference compared to the baseline in 2050

|  | CAN | USA | BRA | OSA | FSU | EU27 | REU | MENA | SSA | CHN | IND | SEA | OAS | ANZ | World |
| --- | --- | --- | --- | --- | --- | --- | --- | --- | --- | --- | --- | --- | --- | --- | --- |
| GDP | 0.03 | 0.01 | -0.10 | -0.03 | 0.06 | 0.10 | 0.04 | 0.05 | 0.06 | -0.01 | 0.06 | 0.00 | 0.14 | 0.05 | 0.03 |
| Land rent | -1.4 | -0.7 | -1.1 | -1.2 | -1.8 | -3.4 | -2.7 | -1.4 | -0.4 | -1.2 | -0.4 | -0.5 | -0.3 | -1.6 | -0.7 |
| Per capita per day quantity of calories | 0.0 | 0.1 | 0.0 | 0.1 | 0.1 | 0.2 | 0.1 | 0.2 | 0.1 | 0.0 | 0.0 | 0.0 | 0.2 | 0.1 | 0.1 |
| Unskilled wages to the cereal price index | 0.86 | 0.67 | -0.22 | 0.16 | 0.82 | 2.02 | 0.93 | 0.52 | 0.31 | 0.07 | 0.35 | 0.03 | 0.44 | 1.08 | 0.34 |

Note: CAN=Canada, USA=United States, BRA=Brazil, OSA=Other Latin America, REU=Other Europe apart from EU27, MENA=Middle-East and North Africa, SSA=Sub-Saharan Africa, CHN=China, IND=India, SEA=South-East Asia, OAS=Other Asia, ANZ=Australia and New Zealand.

Table A8b. Percentage change in indicators by region in Scenario 2 (all countries shocked), percentage difference compared to the baseline in 2050

|  | CAN | USA | BRA | OSA | FSU | EU27 | REU | MENA | SSA | CHN | IND | SEA | OAS | ANZ | World |
| --- | --- | --- | --- | --- | --- | --- | --- | --- | --- | --- | --- | --- | --- | --- | --- |
| GDP | 0.06 | 0.03 | -0.05 | 0.03 | 0.15 | 0.01 | 0.01 | 0.15 | 0.47 | 0.26 | 0.67 | 0.07 | 0.95 | 0.08 | 0.17 |
| Land rent | -3.4 | -2.2 | -2.3 | -2.8 | -3.2 | -4.4 | -3.6 | -3.1 | -3.1 | -5.0 | -2.4 | -1.6 | -1.7 | -4.4 | -2.9 |
| Per capita per day quantity of calories | 0.3 | 0.4 | 0.0 | 0.2 | 0.3 | 0.2 | 0.2 | 0.4 | 0.5 | 0.4 | 0.3 | 0.1 | 0.4 | 0.4 | 0.3 |
| Unskilled wages to the cereal price index | 0.60 | 0.40 | -0.78 | -0.46 | 0.45 | 1.39 | 0.93 | 0.08 | -0.24 | -0.47 | -0.09 | -0.46 | 0.22 | 0.72 | -0.19 |

Note: CAN=Canada, USA=United States, BRA=Brazil, OSA=Other Latin America, REU=Other Europe apart from EU27, MENA=Middle-East and North Africa, SSA=Sub-Saharan Africa, CHN=China, IND=India, SEA=South-East Asia, OAS=Other Asia, ANZ=Australia and New Zealand.

Table A9a. Percentage change in trade volume indicators by region in Scenario 1 (high-income countries shocked), percentage difference compared to the baseline in 2050

|  | | CAN | USA | BRA | OSA | FSU | EU27 | REU | MENA | SSA | CHN | IND | SEA | OAS | ANZ |
| --- | --- | --- | --- | --- | --- | --- | --- | --- | --- | --- | --- | --- | --- | --- | --- |
| Exports volume | Primary agriculture | 0.2 | 1.2 | -1.3 | -1.2 | -0.9 | 5.8 | -0.4 | -2.3 | -1.6 | -1.6 | -2.1 | -1.6 | -1.7 | 0.9 |
|  | Food processing | 0.3 | 0.3 | -0.8 | -0.2 | -0.1 | 0.8 | -0.3 | 0.3 | -0.3 | -0.3 | -0.2 | -0.3 | 0.0 | 0.5 |
|  | Aggregated agri-food | 0.3 | 0.6 | -1.2 | -0.7 | -0.5 | 2.2 | -0.3 | -0.6 | -1.1 | -0.8 | -0.6 | -0.5 | -0.2 | 0.7 |
| Import volume | Primary agriculture | 0.4 | -0.5 | 0.2 | 0.5 | 0.6 | -3.3 | 0.6 | 1.0 | 1.9 | 0.2 | 0.5 | 0.3 | 0.7 | -0.6 |
|  | Food processing | 0.0 | 0.1 | 0.2 | 0.4 | 0.2 | -0.7 | 0.3 | 0.0 | 0.3 | 0.2 | 0.1 | 0.2 | 0.1 | 0.1 |
|  | Aggregated agri-food | 0.1 | -0.1 | 0.2 | 0.4 | 0.4 | -1.8 | 0.4 | 0.4 | 0.5 | 0.2 | 0.3 | 0.2 | 0.4 | 0.0 |

Note 1: CAN=Canada, USA=United States, BRA=Brazil, OSA=Other Latin America, REU=Other Europe apart from EU27, MENA=Middle-East and North Africa, SSA=Sub-Saharan Africa, CHN=China, IND=India, SEA=South-East Asia, OAS=Other Asia, ANZ=Australia and New Zealand.

Note 2: Trade flows in Table 3a refer to aggregated trade to the rest of the world, excluding intra-regional trade.

Table A9b. Percentage change in trade volume indicators by region in Scenario 2 (all countries shocked), percentage difference compared to the baseline in 2050

|  | | CAN | USA | BRA | OSA | FSU | EU27 | REU | MENA | SSA | CHN | IND | SEA | OAS | ANZ |
| --- | --- | --- | --- | --- | --- | --- | --- | --- | --- | --- | --- | --- | --- | --- | --- |
| Exports volume | Primary agriculture | 0.1 | 1.5 | -0.2 | 1.0 | 0.5 | -0.6 | -2.7 | -1.3 | 2.5 | 1.6 | 1.0 | -2.5 | 0.0 | 1.4 |
|  | Food Processing | 0.3 | -0.1 | -0.4 | 0.4 | -0.4 | -0.5 | -0.9 | 0.6 | 1.7 | 1.5 | 2.0 | -0.4 | 1.2 | 0.4 |
|  | Aggregated agri-food | 0.2 | 0.5 | -0.3 | 0.7 | 0.1 | -0.5 | -1.2 | 0.0 | 2.2 | 1.6 | 1.8 | -0.8 | 1.0 | 0.8 |
| Import volume | Primary agriculture | 1.1 | -0.2 | 0.7 | -0.5 | 0.3 | 1.2 | 0.5 | 0.3 | -1.0 | -0.2 | -0.1 | 1.2 | 0.7 | -0.9 |
|  | Food Processing | 0.0 | 0.3 | 0.7 | 0.0 | 0.4 | 0.7 | 0.5 | -0.1 | -0.1 | -0.6 | -0.6 | 0.6 | -0.6 | 0.3 |
|  | Aggregated agri-food | 0.4 | 0.1 | 0.7 | -0.2 | 0.4 | 0.9 | 0.5 | 0.1 | -0.3 | -0.4 | -0.4 | 0.9 | 0.0 | 0.1 |

Note 1: CAN=Canada, USA=United States, BRA=Brazil, OSA=Other Latin America, REU=Other Europe apart from EU27, MENA=Middle-East and North Africa, SSA=Sub-Saharan Africa, CHN=China, IND=India, SEA=South-East Asia, OAS=Other Asia, ANZ=Australia and New Zealand.

Note 2: Trade flows in Table 3a refer to aggregated trade to the rest of the world, excluding intra-regional trade.

**S6. Parameters of agricultural productivity in the MAGNET model**

Table A10a. Growth of agricultural productivity due to accelerating agricultural R&D transfer in high-income countries (Scenario 1)

|  | Growth of the aggregated factor-augmenting technical change ($\boldsymbol{afa\_RD}_{\mathbf{j,i}}$) % | | Growth of the composite factor-augmenting technical change ($\mathbf{afa}_{\mathbf{e,j,i}}$) % | |
| --- | --- | --- | --- | --- |
|  | 2030 | 2050 | 2030 | 2050 |
| CAN | 2.4 | 0.5 | 0.7 | 0.1 |
| USA | 2.4 | 1.0 | 0.8 | 0.4 |
| BRA | 0.0 | 0.0 | 0.0 | 0.0 |
| OSA | 0.0 | 0.0 | 0.0 | 0.0 |
| FSU | 0.0 | 0.0 | 0.0 | 0.0 |
| EU27 | 15.2 | 2.9 | 7.5 | 1.6 |
| REU | 14.3 | 0.3 | 7.1 | 0.2 |
| MENA | 0.0 | 0.0 | 0.0 | 0.0 |
| SSA | 0.0 | 0.0 | 0.0 | 0.0 |
| CHN | 0.0 | 0.0 | 0.0 | 0.0 |
| IND | 0.0 | 0.0 | 0.0 | 0.0 |
| SEA | 0.0 | 0.0 | 0.0 | 0.0 |
| OAS | 0.0 | 0.0 | 0.0 | 0.0 |
| ANZ | 3.6 | 0.9 | 1.7 | 0.4 |
| WORLD | 2.5 | 0.4 | 0.9 | 0.2 |

Note: CAN=Canada, USA=United States, BRA=Brazil, OSA=Other Latin America, REU=Other Europe apart from EU27, MENA=Middle-East and North Africa, SSA=Sub-Saharan Africa, CHN=China, IND=India, SEA=South-East Asia, OAS=Other Asia, ANZ=Australia and New Zealand.

Note 2: The calculation of ${afa\_RD}_{j,i}$ and ${afa}_{e,j,i}$ is based on Equations A1 and A2 in the appendix

Table A10b. Growth of agricultural productivity due to accelerating agricultural R&D transfer in all the countries (Scenario 2)

|  | Growth of the aggregated factor-augmenting technical change ($\boldsymbol{afa\_RD}_{\mathbf{j,i}}$) % | | Growth of the composite factor-augmenting technical change ($\mathbf{afa}_{\mathbf{e,j,i}}$) % | |
| --- | --- | --- | --- | --- |
|  | 2030 | 2050 | 2030 | 2050 |
| CAN | 5.0 | 0.9 | 1.4 | 0.3 |
| USA | 5.0 | 2.0 | 1.7 | 0.7 |
| BRA | 0.4 | 1.9 | 0.2 | 1.0 |
| OSA | 0.4 | 1.7 | 0.2 | 1.0 |
| FSU | 6.2 | 1.0 | 3.3 | 0.5 |
| EU27 | 11.2 | 1.3 | 5.5 | 0.7 |
| REU | 10.5 | 0.2 | 5.2 | 0.1 |
| MENA | 6.2 | 1.1 | 3.4 | 0.6 |
| SSA | 6.2 | 1.1 | 4.3 | 0.8 |
| CHN | 8.7 | 1.5 | 5.6 | 1.0 |
| IND | 6.2 | 1.0 | 4.3 | 0.7 |
| SEA | 6.0 | 0.6 | 3.4 | 0.3 |
| OAS | 6.1 | 1.0 | 3.9 | 0.7 |
| ANZ | 7.4 | 1.6 | 3.4 | 0.8 |
| WORLD | 6.0 | 1.2 | 3.8 | 0.8 |

Note: CAN=Canada, USA=United States, BRA=Brazil, OSA=Other Latin America, REU=Other Europe apart from EU27, MENA=Middle-East and North Africa, SSA=Sub-Saharan Africa, CHN=China, IND=India, SEA=South-East Asia, OAS=Other Asia, ANZ=Australia and New Zealand.

Note 2: The calculation of ${afa\_RD}_{j,i}$ and ${afa}_{e,j,i}$ is based on Equations A1 and A2 in the appendix.
